# Pleural fluid ADA activity in tuberculous pleurisy can be low in elderly, critically ill patients with multi-organ failure

**DOI:** 10.1186/s12890-020-1049-6

**Published:** 2020-01-14

**Authors:** Sae Byol Kim, Beomsu Shin, Ji-Ho Lee, Seok Jeong Lee, Myoung Kyu Lee, Won-Yeon Lee, Suk Joong Yong, Sang-Ha Kim

**Affiliations:** Department of Internal Medicine, Yonsei University Wonju College of Medicine, 20 Ilsan-ro, Wonju, Gangwon 26326 Republic of Korea

**Keywords:** Adenosine deaminase, Tuberculosis, Pleural effusion

## Abstract

**Background:**

Adenosine deaminase (ADA) activity is typically elevated in patients with tuberculous pleural effusion (TPE), but low ADA has occasionally been reported in patients with TPE. The characteristics of these patients are not well-known, and erroneous exclusion of the possibility of TPE can result in a delayed diagnosis. This study investigated the characteristics of patients with TPE who had low ADA activity.

**Methods:**

We retrospectively reviewed patients with microbiologically or pathologically confirmed TPE between 2012 to 2018 in a tertiary hospital in South Korea. Patients were categorised into two groups: high ADA (≥40 IU/L) and low ADA (< 40 IU/L). Clinical characteristics and Sequential Organ Failure Assessment (SOFA) scores were compared between groups.

**Results:**

A total of 192 patients with TPE were included; 36 (18.8%) had ADA < 40 IU/L with a mean ADA activity level of 20.9 (±9.2) IU/L. Patients with low ADA were older (75.3 vs. 62.0 years, *p* < 0.001) and had a lower mean lymphocyte percentage (47.6% vs. 69.9%, *p* < 0.001) than patients with high ADA. Patients in the low ADA group had a significantly higher mean SOFA score (2.31 vs. 0.68, *p* < 0.001), and patients with organ dysfunction were significantly more common in the low ADA group (*p <* 0.001). Patients with 2 or ≥ 3 organ dysfunctions constituted 19.4 and 13.9% of the patients in the low ADA group, whereas they constituted 7.1 and 1.3% of the patients in the high ADA group (*p <* 0.001). Multivariate logistic regression analyses showed that older age (odds ratio = 1.030, 95% confidence interval 1.002–1.060, *p* = 0.038) and a higher SOFA score (odds ratio = 1.598, 95% confidence interval 1.239–2.060, *p* < 0.001) were significantly associated with low ADA activity in patients with TPE.

**Conclusions:**

ADA activity can be low in patients with TPE who are elderly, critically ill, and exhibit multiorgan failure. Low ADA activity cannot completely exclude the diagnosis of TPE, and physicians should exercise caution when interpreting pleural fluid exams.

## Background

Tuberculous pleural effusion (TPE) is a main cause of pleural effusion [1]. The proportion of TPE among all pleural effusions varies widely in accordance with the burden of tuberculosis worldwide. It is less than 10% in countries with low tuberculosis burden, whereas it is reportedly greater than 40% in countries with high tuberculosis burden [2]. Because of the inherent low sensitivity and delay in timing of the demonstration of *Mycobacterium tuberculosis* (MTB) in pleural fluid, a presumptive diagnosis of TPE is frequently made in patients who exhibit lymphocyte-dominant exudative pleural effusion with a high level of adenosine deaminase (ADA) activity [3]. The diagnostic sensitivity and specificity of ADA in TPE are reportedly approximately 90% [4, 5]. Because the negative predictive value of ADA is as high as 99.9% even in countries with low tuberculosis burden [5], a low level of ADA activity is frequently considered an exclusion criterion for the diagnosis of TPE in clinical practice. A few cases of low ADA activity have been reported in patients with TPE, but they showed increased activity in a second sample [6].

The burden of tuberculosis remains high in South Korea, and its incidence was estimated to be 70 per 100,000 in 2017 [7]. A low level of ADA activity in TPE is occasionally observed, particularly in countries with high tuberculosis burden. Falsely normal ADA activity has been reported in up to 7% of patients with TPE [8]. One study analysed 182 patients with TPE and reported that 22 demonstrated ADA activity of less than 40 IU/L [9]. An early diagnosis of TPE in the absence of microbiological evidence is made based on laboratory findings and clinical context. Without consideration of the possibility of low ADA activity in TPE, a diagnosis of TPE can be erroneously excluded. Thus, the present study investigated the characteristics of patients with TPE who had low ADA activity.

## Methods

### Study population

We retrospectively analysed patients with TPE who were admitted to Wonju Severance Christian Hospital, a local tertiary hospital in South Korea, between 1 January 2012 and 31 December 2018. All patients who presented with undiagnosed pleural effusion and underwent pleural fluid analyses were screened; patients with a final diagnosis of TPE were included in the analyses. The diagnostic criteria for TPE were as follows: (1) growth of MTB from pleural fluid, or polymerase chain reaction (PCR) results indicative of the presence of MTB in pleural fluid; (2) demonstration of tuberculous granuloma in a pleural biopsy specimen with caseous necrosis, positive Ziehl-Nielsen staining, or PCR results indicative of the presence of MTB; (3) growth of MTB from a respiratory specimen (sputum or bronchial wash) or PCR results indicative of the presence of MTB in a respiratory specimen, as well as the absence of other definite causes of pleural effusion. Patients with a presumptive diagnosis of TPE without microbiologic or pathologic evidence were excluded from the analyses.

### Data collection

Pleural fluid analyses and measurements of total ADA and ADA2 isoenzyme activities were performed in every patient. The cut-off value for low ADA was < 40 IU/L, which is the most widely accepted value in the literature [4]. Data were collected from medical records regarding patient characteristics such as age, sex, previous medical history, and smoking status. Total leukocyte count in the pleural fluid was assessed, as were the percentages of polymorphonuclear leukocytes (PMNs) and lymphocytes in total fluid leukocytes. The Sequential Organ Failure Assessment (SOFA) score was calculated on admission to assess organ dysfunction, which ranges from 0 to 4 for each of six organ systems: respiratory, coagulation, liver, cardiovascular, central nervous system, and renal [10]. SOFA score ≥ 1 for each system was considered indicative of the relevant organ dysfunction. The number of organ dysfunctions was considered the number of organ systems involved (i.e., with SOFA score ≥ 1). Clinical data were also reviewed regarding the route of admission (outpatient clinic or emergency department), incidence of intensive care unit (ICU) stay, and 30-day mortality.

### Statistical analyses

Statistical analyses were performed using SPSS software (version 23.0; SPSS Inc., Chicago, IL, USA). Variables in the high and low ADA groups were compared using the independent t-test and chi-square test. Data shown are mean values ± standard deviations, or the numbers of patients (percentages). Multivariate logistic regression analyses were performed to investigate the associations of clinical variables with low ADA activity in patients with TPE. A *p*-value < 0.05 was considered statistically significant.

## Results

**Fig. 1:**
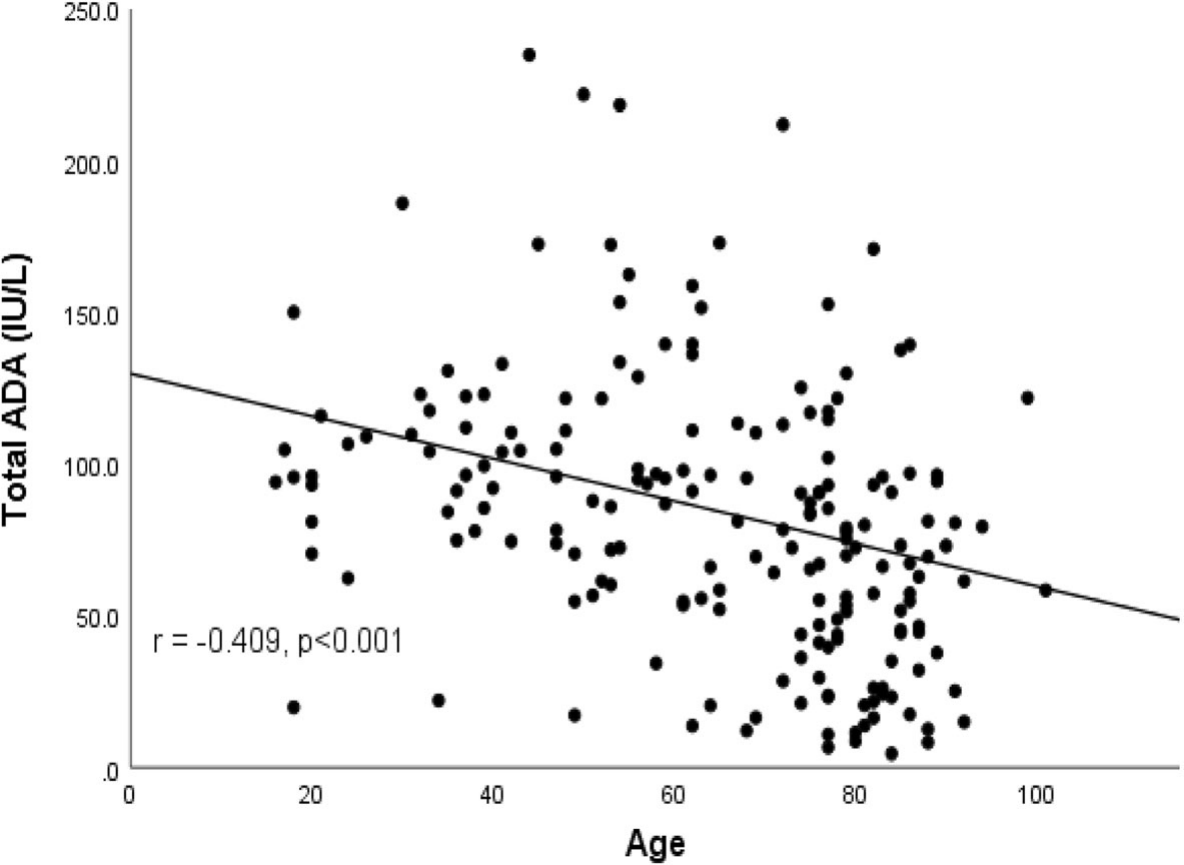
Scatter diagram showing an inverse correlation between age and total ADA activity in patients with TPE. ADA = adenosine deaminase; TPE = tuberculous pleural effusion

A total of 192 patients with TPE were included in the analyses; patients’ baseline characteristics are shown in Table 1. Overall, the mean age of the patients was 64.5 years; 67.7% of the patients were male, and 22.4% were current smokers. 21.4, 42.2, and 8.9% of the patients had diabetes mellitus, hypertension, and a history of previous tuberculosis treatment, respectively. Among the 192 patients, 36 (18.8%) had an ADA activity of less than 40 IU/L; their mean ADA activity was 20.9 ± 9.2 IU/L. Patients in the low ADA group were older than those in the high ADA group (75.3 vs. 62.0 years, *p* < 0.001), with a correlation coefficient of − 0.409 (*p* < 0.001) (Fig. 1). More patients in the low ADA group had hypertension (58.3% vs. 38.5%, *p* = 0.030), whereas other co-morbidities and smoking statuses were not significantly different between the two groups. Pleural fluid analyses showed lymphocyte predominance (65.9%). Patients in the low ADA group had a lower mean lymphocyte proportion than those in the high ADA group (47.6% vs. 69.9%, *p* < 0.001); they also had a higher mean number of PMNs (33.2% vs. 23.7%, *p* = 0.153), but this difference was not statistically significant. The mean total ADA activity was much lower in the low ADA group, whereas the mean activity ratio (%) of the ADA2 isoenzyme was consistently high in both groups (63.4% in the high ADA group vs. 65.4% in the low ADA group, *p* = 0.355).

**Table 1:** Baseline Characteristics

	Total (*n* = 192)	High ADA (≥40 IU/L) (*n* = 156)	Low ADA (< 40 IU/L) (*n* = 36)	*p*-value
Age (years)	64.5 (±20.5)	62.0 (±20.7)	75.3 (±15.4)	< 0.001
Sex (% male)	130 (67.7%)	110 (70.5%)	20 (55.6%)	0.084
Smoking status				0.322
Never smoker	103 (53.6%)	83 (53.2%)	20 (55.6%)	
Current smoker	43 (22.4%)	38 (24.4%)	5 (13.9%)	
Former smoker	46 (24.0%)	35 (22.4%)	11 (30.6%)	
DM	41 (21.4%)	33 (21.2%)	8 (22.2%)	0.888
HTN	81 (42.2%)	60 (38.5%)	21 (58.3%)	0.030
History of TB	17 (8.9%)	14 (9.0%)	3 (8.3%)	0.903
Pleural fluid analyses				
WBC (/μL)	4,141 (±15,970)	4362 (±17,371)	3,168 (±7,229)	0.708
PMNs (%)	25.4 (±31.2)	23.7 (±29.7)	33.2 (±36.6)	0.153
Lymphocyte (%)	65.9 (±31.2)	69.9 (±29.4)	47.6 (±32.9)	< 0.001
ADA (IU/L)	84.6 (±54.1)	99.3 (±49.3)	20.9 (±9.2)	< 0.001
ADA2 (% of total ADA)	63.7 (±11.7)	63.4 (±12.0)	65.4 (±10.2)	0.355

*ADA* total adenosine deaminase, *ADA2* adenosine deaminase isoenzyme 2, *DM* diabetes mellitus, *HTN* hypertension, *TB* tuberculosis, *WBC* white blood cell, *PMNs* polymorphonuclear leukocytes

**Fig. 2:**
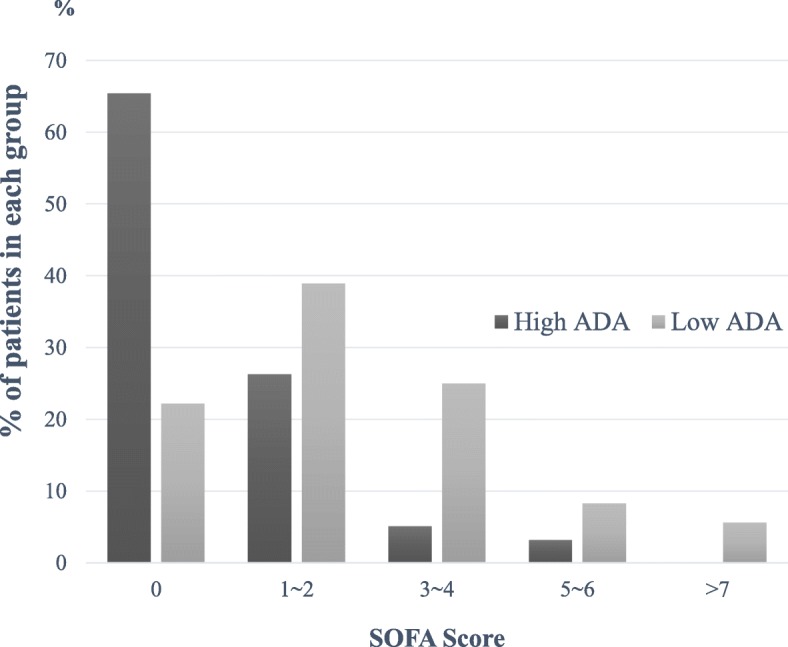
SOFA scores in patients in the high and low ADA groups. ADA = adenosine deaminase; SOFA = Sequential Organ Failure Assessment

**Fig. 3:**
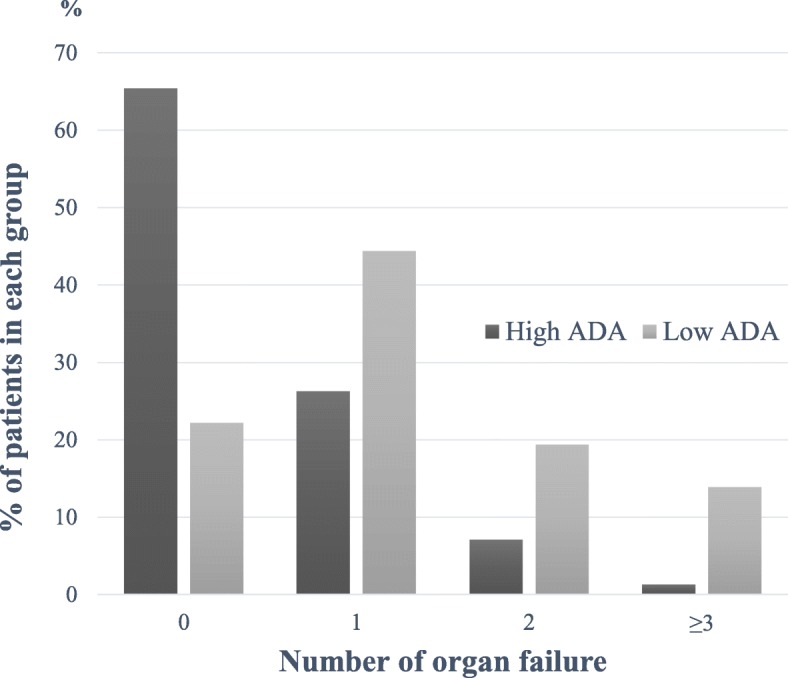
Numbers of organs involved in patients in the high and low ADA groups. ADA = adenosine deaminase

More patients had organ dysfunction in the low ADA group (Table 2). More patients in the low ADA group had respiratory, coagulation, cardiovascular, central nervous system, and renal dysfunctions than in patients in the high ADA group (36.1% vs. 15.4%, *p* = 0.004; 11.1% vs. 1.3%, *p* = 0.002; 19.4% vs. 1.9%, *p* < 0.001; 11.1% vs. 1.9%, *p* = 0.008; 36.1% vs. 14.7%, *p* = 0.003, respectively). Patients in the low ADA group had a significantly higher mean SOFA score (2.31 vs. 0.68, *p* < 0.001). In the low ADA group, 22.2% of patients had a SOFA score of 0, 38.9% had 1 or 2, 25.0% had 3 or 4, 8.3% had 5 or 6, and 5.6% had SOFA score ≥ 7; in the high ADA group, 65.4% of patients had a SOFA score of 0, 26.3% had 1 or 2, 5.1% had 3 or 4, 3.2% had 5 or 6, and none had SOFA score ≥ 7 (Fig. 2). In total, 65.4% of patients in the high ADA group had no organ dysfunction, whereas 77.8% of patients in the low ADA group had one or more organ dysfunctions. Multiorgan involvement was significantly more common in the low ADA group (*p* < 0.001). Patients with 2 or ≥ 3 organ dysfunctions constituted 19.4 and 13.9% of the patients in the low ADA group, whereas they constituted 7.1 and 1.3% of the patients in the high ADA group (Fig. 3). More patients in the low ADA group were admitted through the emergency department (83.3% vs. 62.2%, *p* = 0.016) and had stayed in the ICU (27.8% vs. 9.6%, *p* = 0.004). Overall mortality within 30 days was higher in the low ADA group than in the high ADA group (13.9% vs. 2.6%, *p* = 0.004).

**Table 2:** Organ dysfunction, SOFA score, and clinical outcomes in the high and low ADA groups

	High ADA (≥40 IU/L) (*n* = 156)	Low ADA (< 40 IU/L) (*n* = 36)	*p*-value
Organ dysfunction (SOFA score ≥ 1)			
Respiratory	24 (15.4%)	13 (36.1%)	0.004
Coagulation	2 (1.3%)	4 (11.1%)	0.002
Liver	14 (9.0%)	7 (19.4%)	0.070
Cardiovascular	3 (1.9%)	7 (19.4%)	< 0.001
CNS	3 (1.9%)	4 (11.1%)	0.008
Renal	23 (14.7%)	13 (36.1%)	0.003
SOFA score	0.68 (±1.24)	2.31 (±2.34)	< 0.001
0	102 (65.4%)	8 (22.2%)	
1–2	41 (26.3%)	14 (38.9%)	
3–4	8 (5.1%)	9 (25.0%)	
5–6	5 (3.2%)	3 (8.3%)	
≥ 7	0 (0.0%)	2 (5.6%)	
Number of organs involved			< 0.001
0	102 (65.4%)	8 (22.2%)	
1	41 (26.3%)	16 (44.4%)	
2	11 (7.1%)	7 (19.4%)	
≥ 3	2 (1.3%)	5 (13.9%)	
ED visit	97 (62.2%)	30 (83.3%)	0.016
ICU stay	15 (9.6%)	10 (27.8%)	0.004
Death (≤30 days)	4 (2.6%)	5 (13.9%)	0.004

*ADA* total adenosine deaminase, *SOFA* Sequential Organ Failure Assessment, *ED* emergency department, *ICU* intensive care unit

Multivariate logistic regression analyses showed that older age (odds ratio = 1.030, 95% confidence interval 1.002–1.060, *p* = 0.038) and a higher SOFA score (odds ratio = 1.598, 95% confidence interval 1.239–2.060, *p* < 0.001) were significantly associated with low ADA activity in patients with TPE (Table 3).

**Table 3:** Associations of clinical variables with low ADA activity (< 40 IU/L) in patients with TPE: multivariate logistic regression analyses

	Odds ratio	95% CI	*p*-value
Age	1.030	1.002–1.060	0.038
Sex (male)	2.067	0.708–6.039	0.184
Smoking status			
Never smoker	1		
Current smoker	0.592	0.184–1.908	0.380
Former smoker	0.677	0.176–2.596	0.569
DM	1.316	0.472–3.672	0.600
HTN	1.220	0.474–3.142	0.680
History of TB	0.958	0.212–4.319	0.955
SOFA score	1.598	1.239–2.060	< 0.001

*ADA* total adenosine deaminase, *TPE* tuberculous pleural effusion, *CI* confidence interval, *DM* diabetes mellitus, *HTN* hypertension, *TB* tuberculosis, *SOFA* Sequential Organ Failure Assessment

## Discussion

We investigated the characteristics of patients with TPE who presented with low ADA activity. Patients in the low ADA group were older than those in the high ADA group. Patients in the low ADA group showed less lymphocyte predominance, and more frequently exhibited a high SOFA score and multiple organ failure. They were more likely to be admitted through the emergency department, stay in the ICU, and exhibit higher 30-day mortality.

Typical manifestations of TPE are well-recognised, including submassive, unilateral pleural effusion with lymphocyte predominance and high ADA [1, 8]. However, atypical presentations of TPE have been reported; falsely normal ADA activity occurs in up to 7% of patients with TPE [4, 8]. A few cases of low ADA activity have previously been reported in patients with TPE. In one study, a single patient with TPE had a low ADA activity of 22 U/L, which increased to 91 U/L in a second sample [11]. Querol et al. reported nine patients with TPE who exhibited low ADA activity (< 43 U/L) and showed that in five of those nine patients, a second sample showed an increase in ADA activity to > 43 U/L [6]. Based on these reports, a second determination of ADA activity is recommended when a clinical suspicion of TPE exists and the level of ADA activity is low.

In 2014, one study investigated factors influencing pleural ADA activity in patients with TPE and reported that pleural ADA activity can be low in elderly and current smokers [9]. Among 182 patients with TPE in that study, 22 patients (12.1%) had low ADA activity (< 40 IU/L). In the current study, 36 patients (18.8%) among 192 had ADA activity of less than 40 IU/L. The proportion of patients with TPE who have low ADA activity may be higher than previously estimated, particularly in countries with a high tuberculosis burden. Understanding the characteristics of these patients will help to interpret the results of pleural fluid analyses and prevent delayed diagnosis of TPE.

Our results showed that patients were older in the low ADA group; notably, there was a negative correlation between age and ADA activity in patients with TPE, which is consistent with the findings of previous studies [9, 12]. The prevalence of hypertension was also higher in the low ADA group, but it is likely confounded by higher mean age in this group and the multivariate regression analysis did not show that hypertension is an independent risk factor. Age-associated immune decline and T-cell immunosenescence are well-recognised [13, 14] and Tay et al. suggested that that a lower ADA cut-off value should be used in elderly patients [12]. TPE commonly occurs as an opportunistic infection in patients with human immunodeficiency virus (HIV) in many countries with high TB and HIV burden, but investigations regarding the optimal diagnostic value for ADA activity in immunocompromised hosts have shown inconsistent results and only included a limited number of patients [15–17]. Further studies are needed to provide useful recommendations for diagnosis of such patients.

Typical TPE manifestation involves lymphocyte predominance. However, up to 10% of tuberculous effusions are neutrophil-dominant, which is thought to reflect an early or acute process of disease [18, 19]. Although PMN predominance did not significantly differ between groups in this study, the lower lymphocyte predominance in the low ADA group may be indirectly related to an early and acute disease process. Patients in the low ADA group also had a higher SOFA score, higher rate of admission through the emergency department, and higher rates of ICU stay and multiple organ involvement, as well as higher mortality. Based on our data, we suggest that cautions should be taken for the subset of patients with early and acute disease processes, in which laboratory findings may differ from those of typical cases.

Increased capillary permeability in TPE is caused by delayed hypersensitivity response to mycobacterial protein, which is released to the pleural cavity by the rupture of subpleural caseous foci. The immunological reaction involves activation of T lymphocytes which stimulate macrophages to perform anti-mycobacterial functions [20]. ADA is a predominant T-lymphocyte enzyme, which has two molecular isotypes, ADA1 and ADA2. The ADA2 isoenzyme is primarily response for the increased total ADA activity in patients with TPE [2]. The initial low value of ADA in patients with TPE is generally thought to increase when a second sample is taken, as previously observed [6, 11]. However, a second measurement of ADA activity was performed in 10 of 32 patients with TPE who had low ADA in this study; only 3 of those patients showed increased ADA activity (i.e., ≥40 IU/L). Falsely negating the possibility of TPE in critically ill patients who have low ADA activity can result in delayed diagnosis and potentially avoidable death.

The diagnostic value of the ADA2 isoenzyme has largely been investigated in prior studies and is slightly superior to that of total ADA with respect to the diagnosis of TPE [21]. However, determining ADA2 is not routinely recommended because of its low additional yield, limited availability, and poor cost-effectiveness [2, 22]. In this study, patients in the low ADA group had a high proportion of ADA2 activity, similar to that of patients in the high ADA group, which implies a similar effect of tuberculosis-related immune reactions. Although this does not confirm a specific diagnostic value with respect to the ADA2 isoenzyme, it suggests that a high index of suspicion for TPE should be maintained in patients who have otherwise unexplainable pleural effusion with low total ADA activity, if they exhibit a high proportion of ADA2 activity. If other evidence supports a diagnosis of TPE, especially in cases of critically ill patients, a trial course of tuberculosis medication might be considered based on the clinical context.

This study had some limitations. Most importantly, it was a retrospective study and some clinical data (e.g., cardiac function, presence of comorbid bacterial pneumonia, and course of renal insufficiency) could not be obtained, due to a lack of medical records or the absence of a standardised evaluation protocol. Therefore, this study only provides data regarding the clinical characteristics of patients with TPE who had low ADA, and further studies are needed to determine the pathophysiology and clinical courses of these patients. Furthermore, investigations regarding the diagnostic value of ADA2 isoenzyme activity may aid in early diagnosis of TPE in unique situations where total ADA activity measurements provide false negative findings.

## Conclusions

ADA activity can be low in patients with TPE who are elderly, critically ill, and exhibit multiorgan failure. Physicians should be aware of the characteristics of these patients and exercise caution when interpreting pleural fluid exams. Low ADA activity cannot completely exclude the diagnosis of TPE.

## Data Availability

The datasets used and analysed during the current study are available from the corresponding author on reasonable request.

## Abbreviations

ADA: Adenosine deaminase
CI: Confidence interval
DM: Diabetes mellitus
ED: Emergency department
HIV: Human immunodeficiency virus
HTN: Hypertension
ICU: Intensive care unit
MTB: *Mycobacterium tuberculosis*
OR: Odds ratio
PCR: Polymerase chain reaction
PMNs: Polymorphonuclear leukocytes
SOFA: Sequential Organ Failure Assessment
TB: Tuberculosis
TPE: Tuberculous pleural effusion
WBC: White blood cell
